# Thromboinflammatory complications of *Bothrops* snakebite envenoming: the case of *B. lanceolatus* endemic to the Caribbean Island of Martinique

**DOI:** 10.3389/fimmu.2025.1625165

**Published:** 2025-09-10

**Authors:** Caroline Rapon, Jonathan Florentin, Fatima Radouani, Prisca Jalta, Florian Negrello, Papa Gueye, Olivier Pierre-Louis, Remi Neviere, Dabor Resiere

**Affiliations:** ^1^ Cardiovascular Research Team (UR5_3 PC2E), University of the French West Indies (Université des Antilles), Fort de France, France; ^2^ Department of Toxicology and Critical Care Medicine, University Hospital of Martinique (CHU Martinique), Fort-de-France, France; ^3^ Department of Research, University Hospital of Martinique (CHU Martinique), Fort-de-France, France; ^4^ Department of Emergency Medicine SAMU 972, University Hospital of Martinique (CHU Martinique), Fort-de-France, France

**Keywords:** Bothrops, envenoming, thrombosis, inflammation, NETs, kallikrein, von Willebrand factor

## Abstract

Snakebite envenoming remains a predominant neglected disease in tropical and subtropical regions, with high rates of morbidity and mortality worldwide. *Bothrops* snakebite envenoming. is characterized by severe injuries at the site of venom injection, which include tissue necrosis, hemorrhage, blistering, and edema. Haemotoxicity is typically attributed to the strong procoagulant state induced by the majority *Bothrops* venoms leading to coagulation factor consumption and incoagulable blood. Concomitantly with this procoagulant state, a complex host response develops in the affected tissues, accompanied by the recruitment of inflammatory and immunocompetent cells, along with the activation of resident cells, and the synthesis of a plethora of pro-inflammatory mediators and damage-associated molecular patterns from injured tissue. An increasing body of evidence suggests that this intricate response is, in fact, related to the well-documented immunothrombosis and thromboinflammation integrated features. Of note, thrombotic complications are extremely rare in *Bothrops* snakebite envenoming. However, in the case of *Bothrops lanceolatus* and *B. caribbaeus*, which are respectively endemic to Martinique and St. Lucia, the absence of overt consumption coagulopathy due to their weak procoagulant effects may be related to the thrombotic effects, as clotting factors are present in the bloodstream by the time the thrombogenic and inflammatory mechanisms are operating in blood vessels. Prior to the era of immunotherapy, *B. lanceolatus* envenoming was associated with thrombotic complications in 25% of cases and was fatal in approximately 10% of cases. This review examines the potential role of thromboinflammation as a mechanism of thrombotic accidents in *B. lanceolatus* snakebite envenoming.

## Highlights

- *Bothrops* snakes are the most common venomous snakes in tropical regions.
- Venoms of *Bothrops* snake have a complex composition of toxins that can trigger a series of systemic effects, including haemotoxicity.
- While thrombosis is a rare feature in *Bothrops* envenoming, *B. lanceolatus* bite can elicit thrombotic complications in 25% of cases.
- Understanding the exact pathological mechanisms involved in *B. lanceolatus* venom-induced thrombotic events are still a challenge for the scientific community.
- Increasing evidence suggests that specific processes involved in thromboinflammation are operative in *B. lanceolatus* envenoming.
- Thromboinflammation may provide new options for the therapeutical approach of *B. lanceolatus* envenoming, such as antithrombin, activated protein C, thrombomodulin, and interleukin inhibitors.

## 1 Introduction

Increasing evidence suggests that specific processes involved in thromboinflammation are operative in *Bothrops* snakebite envenoming. The terms “immunothrombosis” and “thromboinflammation” refer to the complex interplay between thrombotic and inflammatory pathways (1, 2) Immunothrombosis has been proposed to describe an innate immune response involving intravascular thrombus formation that can lead to the recognition, containment, and destruction of pathogens (1, 3–5). Thromboinflammation is associated with specific pathways that operate through mechanisms involving platelets, leukocytes and immunocompetent cells, and the contact kinin system (3, 6–9). Thromboinflammation is increasingly recognized in several pathologies, including infection and sepsis, as well as stroke, deep vein thrombosis, and myocardial infarction (3, 6–13). Thrombo-inflammatory pathways can exacerbate inflammation and immune cell interactions, eventually leading to vascular occlusion, tissue ischemia, and ultimately irreversible organ damage (1, 3, 4, 6, 9, 13).

The procoagulant components of *Bothrops* venoms can cause intravascular coagulation, in most cases can induce a consumption coagulopathy, which results in defibrinogenation and incoagulability, as reflected by abnormal blood clotting tests (14). Envenomed patients present increased prothrombin time (PT) and activated partial thromboplastin time (aPTT), with low level of fibrinogen. Extensive experimental, clinical, and laboratory data underpinned the fact that *Bothrops* snakebite envenoming also elicits a pro-inflammatory state, along with multiple blood cell activation (15–18). However, despite procoagulant state and pro-inflammatory activation, thrombotic complications are extremely rare in *Bothrops* sp. snakebite envenoming, except for those associated with *B. lanceolatus* and *B. caribbaeus* snakes. *B. lanceolatus* and *B. caribbaeus* are endemic to Martinique and St. Lucia, respectively and genetically close (19–25). Better known as” trigonocephalus” or “fer-de-lance”, the *Bothrops lanceolatu*s (Bonnaterre, 1790) is a species of snake belonging to the *Crotalinae* subfamily and the *Vipiridae* family, just like the *Bothrops caribbaeus* (Garman, 1887).

Prior to the era of immunotherapy, *B. lanceolatus* envenoming was associated with thrombotic complications in 25% of cases and was fatal in approximately 10% of cases (20–22). The proposed mechanism of these thrombotic events has been related to the combination of two processes: a weak procoagulant effect of *B. lanceolatus* and *B. caribbaeus* venoms, which does not induce coagulation factor consumption, along with the simultaneous potent activation of thrombogenic and inflammatory processes operating in blood vessels (23, 26–30).

The question of whether the thrombogenic and inflammatory processes induced by *B. lanceolatus* would be considered a manifestation of thromboinflammation has not been explored. This review provides an update to gain insight into the pathophysiological mechanisms involved in thrombo-inflammatory processes associated with *Bothrops* snakebite envenoming. We will discuss the central role of platelet activation, recruitment of peripheral leukocytes and immunocompetent cells, and activation of the contact kinin system. In addition, we will investigate whether thromboinflammation may represent a valuable mechanism of thrombotic accidents in *Bothrops* snakebite envenoming.

## 2 Overview of the toxic effects of *Bothrops* venoms

### 2.1 Local effects

The local effects of *Bothrops* snakebite envenoming are characterized by an intense inflammatory response, a consequence of the direct and indirect action of the venom toxins on the tissues (31). The toxins can directly activate leukocyte receptors, such as Toll-like receptors (TLRs), recognizing venom-associated molecular patterns (VAMPs), and immunological soluble molecules (32–34). In addition, venom toxin-induced tissue damage results in the release of damage-associated molecular patterns (DAMPs), which also contribute to inflammation (35). Direct activation of the complement system by the toxins represents another important mechanism in the pathogenesis of local inflammation (36). The clinical features of *Bothrops* envenomation are well-established, typically presenting with pain, edema, blistering, ecchymosis, local hemorrhage, and, in severe cases, compartment syndrome and necrosis (37–49).

### 2.2 Systemic effects

*Bothrops* venom also induces systemic changes, characterized mainly by coagulation disorders. These can be caused by the direct action of the toxins on coagulation factors, activating the coagulation cascade, leading to a state of blood incoagulability, and by the direct action on platelets, causing platelet death, platelet activation, or cleavage of platelet activation receptors (50). These mechanisms result in platelet consumption, and consequently thrombocytopenia. Together, these factors favor the development of hemorrhage, which can lead to death if not promptly controlled. Thrombotic microangiopathy (TMA) is another systemic manifestation, but less common. TMA appears to be triggered by thrombin generation followed by fibrin formation and deposition in the vascular bed, which are involved in microangiopathic hemolytic anemia and blood vessel wall damage in the micro-circulation. TMA hence carries a risk of organ damage and failure (51–53).

Renal dysfunction following *Bothrops* envenomation may result from dysregulation of the coagulation cascade, direct nephrotoxicity of venom components, systemic hemodynamic alterations such as hypotension, and, in certain cases, the development of thrombotic microangiopathy (TMA) (15, 18, 54–59). Nonetheless, clinical data indicate that the predominant mechanism of acute kidney injury (AKI) in human envenomation cases is closely associated with coagulation disturbances. AKI has been reported in patients exhibiting prolonged activated partial thromboplastin time (aPTT), hemorrhagic manifestations, elevated lactate dehydrogenase (LDH) levels, and evidence of TMA (23, 60, 61).

## 3 *Bothrops* venom induced procoagulant effects and thrombotic events

*Bothrops* venom has been observed to have a range of effects, including thrombotic, procoagulant, and inflammatory properties (15–18). The genus *Bothrops* is the most reported genus responsible for snakebites in South America. Of these, *B. atrox* (Linnaeus, 1758) is the species most frequently involved in cases of life-threatening envenoming in humans (17). In the French Departments of America, *B. atrox* is the predominant species involved in envenoming in French Guiana, whereas *B. lanceolatus* is the sole venomous snake present in Martinique, where it is endemic (23–25, 62). The biological active toxins responsible for these features include SVMPs, serine proteinases (SVSPs), phospholipases A_2_, C-type lectin-like toxins, disintegrins, and *L*-amino acid oxidases (15–18). *Bothrops* snakebite envenoming causes significant local tissue damage and systemic manifestations, including coagulopathies, bleeding, and hemorrhage related to enzymatic degradation and rupture of vessel walls (16, 18).

### 3.1 Procoagulant effects of Bothrops venoms

Envenomed patients typically display increased prothrombin time and activated partial thromboplastin time, along with low level of fibrinogen. Procoagulant toxins derived from *Bothrops* venom have been observed to activate a number of coagulation factors, including factor Factors II, V, VII, X, XIII II (26–28, 63, 64). Factors involved in the intrinsic pathway (factors VIII, IX, XI, XII) are less frequently reduced (26–28, 63, 64). Activation of the coagulation cascade ultimately results in the generation of intravascular thrombin. From a biological standpoint, one of the most notable distinctions between *B. lanceolatus* and *B. caribbaeus*, in comparison to other *Bothrops* species, pertains to their respective procoagulant activities. The procoagulant activities of *Bothrops* venoms elicit the conversion of prothrombin to thrombin, which is responsible for consumptive coagulopathy. The majority of *Bothrops* venoms are capable of activating thrombin without the involvement of cofactors such as calcium and phospholipids (26–28, 30).

Previous research has produced inconsistent findings on the procoagulant effects of *B. lanceolatus* venom (27, 29, 30, 65–69). Early studies suggested that the venom lacked procoagulant or defibrinogenating activity, as it failed to induce clot formation in citrated human plasma (65, 67, 69). However, these *in vitro* experiments assessed coagulation in citrated plasma without added calcium or phospholipids—key cofactors that modulate procoagulant activity. More recent thrombo-elastography studies using human plasma or whole blood have shown that *B. lanceolatus* venom can exhibit procoagulant effects when sufficient calcium and phospholipids are present (27, 29, 30, 66, 68). In brief, the procoagulant activity of *B. lanceolatus* venom appears weaker than that of other *Bothrops* venoms. This activity is entirely calcium-dependent, with a minor reliance on phospholipids (27). Unlike typical procoagulant *Bothrops* venoms—which act by directly activating thrombin (via prothrombin conversion) or indirectly by activating upstream zymogens like factor X (26)—*B. lanceolatus* venom displays a pseudo-coagulant effect. This results in fragile, unstable fibrin clots that degrade quickly (30), possibly explaining its limited procoagulant activity in citrated plasma.

### 3.2 *Bothrops* venom can induce thrombotic complications

Thrombotic complications induced by *Bothrops* venoms are less common than hemorrhagic complications in Amazonian *Bothrops* snakebite envenoming (26). Only a limited number of cases of ischemic strokes have been documented so far (**Supplementary Table 1** summarized the reported thrombotic complications involved in *Bothrops* sp. envenoming).

In contrast to with the typical hemorrhagic profile, venoms of *Bothrops*, such as *B. lanceolatus* (endemic to Martinique), *B. caribbaeus* (endemic to St. Lucia, located approximately 40 km south of Martinique), and *B. atrox* can induce a thrombotic biological profile, which may result in cerebral, myocardial, and pulmonary infarctions (20–22). Prior to the advent of immunotherapy, *B. lanceolatus* envenoming was linked to systemic thrombotic complications in approximately 30% of cases and was fatal in approximately 10% of cases (20–22). Previous studies failed to identify a distinctive proteomic profile between *B. lanceolatus* and *B. caribbaeus* venoms compared with other *Bothrops* venoms (27, 29, 65). It has been, however, demonstrated that enzymatic or non-enzymatic proteins present in *B. lanceolatus* and *B. atrox* venoms may differ in their peptide sequences, which could be responsible for the different biological effects observed, namely hemorrhagic versus thrombotic profiles (29).

Laboratory test abnormalities have been documented regarding activated partial thromboplastin time (aPTT), prothrombin time (PT), prothrombin activity, International Normalized Ratio (INR), fibrinogen consumption, fibrin degradation product increase, thrombocytopenia, along with anemia and leukocytosis (21, 22, 70–79). Furthermore, fibrinogen is a determinant of blood viscosity and platelet activation, also playing a role in the inflammatory process, and is considered a marker related to ischemic stroke (80, 81). Consistent evidence has shown that high levels of fibrinogen may increase the risk of ischemic stroke (82). However, in the case of *Bothrops* snakebites, hypofibrinogenemia occurs as the result of the action of toxins, which are capable of directly cleaving the chains of fibrinogen releasing fibrinopeptide A and traces of fibrinopeptide B (83). In addition, fibrinogen may be also decreased or consumed as the result of coagulation cascade activation. In this regard, toxins from *B*. *atrox*, *B*. *caribbaeus*, *B*. *lanceolatus* and *B*. *marajoensis* (snakes with reports of snakebite envenoming with thrombotic complications) can activate the coagulation cascade via the intrinsic, extrinsic or common pathway. *B. atrox* venom activates factors II, X, XII and V, and increases the procoagulant activity of factor VIII, which, as a result, leads to the generation of intravascular thrombin and hypofibrinogenemia (84–86). In the case of *B. lanceolatus* venom, toxins that may alter coagulation factor activation have not been previously isolated and characterized. It is known, however, that *B. lanceolatus* venom can induce the formation of fibrin in plasma and in purified human fibrinogen, indicating activity similar to thrombin, as well as the degradation of fibrinogen (30). Similarly, *B. caribbaeus* venom can hydrolyze fibrinogen *in vitro* resulting in hypofibrinogenemia and increased levels of fibrin/fibrinogen degradation products *in vivo*, but no increase in D-dimer levels (87). In acute ischemic stroke and infarcts related to *B. lanceolatus* snakebite, a decrease in aPTT and an increase in PT have been observed. Thrombotic complications with (or without) hemorrhagic transformations triggered by *B*. *atrox* venom involve an increase in PT. It is noteworthy that reduced aPTT has been associated with ischemic stroke, severity and neurological worsening (88), although other studies do not corroborate this (89). Of note, the specific pathogenic mechanisms and toxins involved in thrombotic complications associated with *B. lanceolatus* envenoming remain unknown. Proposed mechanisms include venom-induced endothelial injury, platelet activation, involvement of von Willebrand factor, and proinflammatory activity of the venom (26, 90).

In addition to interspecies differences, intra-species factors such as age, gender, geographic location, diet, and captivity conditions may alter *Bothrops* venom composition (29, 91, 92). Accidents with juvenile *Bothrops* cause higher incidence of hemorrhage and coagulation disorders than snakebite with adult snakes, while the latter inflict less inflammation and more severe local tissue damage (93, 94). In the case of *B. lanceolatus*, thrombotic complications are more frequently observed in patients bitten by juvenile snakes (i.e., small snakes of less than 70 cm in length), while envenoming by adult snakes produce more swelling (95). In *Bothrops* sp., ontogenetic variation refers to changes in venom composition as the snake progresses from juvenile to adult stages, often correlating with shifts in diet and prey type (93–97). Sexual variation, conversely, denotes differences between male and female venom, potentially linked to physiological or behavioral dimorphism. Ontogenetic and/or sexual variations have been documented in several *Bothrops* snakes, such as *B. leucurus (*Wagler, 1824*)*, *B. pauloensis* (Amaral, 1925), *B. jararaca*, *B. jararacussu*, *B. moojeni* and *B. atrox* (93–99). These researchers consistently demonstrate that venom from *Bothrops* juvenile snakes often exhibit higher coagulotoxicity with procoagulant activities, whereas venom from *Bothrops* adult snakes display increased hemorrhagic and proteolytic activities. Likewise, previous reports have indicated sexual variations in protein levels, like higher disintegrins in female *B. atrox* and higher serine protease effects in *B. moojeni*, which may influence venom coagulant effects (99, 100).

## 4 Proinflammatory effects of *Bothrops* venoms

### 4.1 The inflammatory process

Inflammation is an immune system response triggered by various factors, such as pathogens, damaged cells, and toxic compounds (101, 102). This process involves the coordinated activation of signaling pathways—primarily NF-κB, MAPK, and JAK-STAT—which regulate the release of inflammatory mediators from resident tissue cells and modulate the activity of blood-derived immune cells (103, 104). Platelets are among the first responders to endothelial injury and microbial threats. Their expression of P-selectin is crucial for forming platelet-leukocyte aggregates, facilitating leukocyte recruitment and their rolling adhesion to the vascular endothelium in the presence of activated platelets (86). Following platelet activation, circulating neutrophils and monocytes rapidly infiltrate injured tissues (105). Meanwhile, resident macrophages and dendritic cells play key roles in tissue immunosurveillance and antigen presentation. The inflammatory response is tightly controlled by mediators such as cytokines, chemokines, vasoactive amines, and eicosanoids, which act both locally and systemically. These molecules are released near the injury site by endothelial cells and resident immune cells (e.g., mast cells and macrophages) during the early inflammatory phase, preceding leukocyte infiltration (101, 102).

### 4.2 Inflammatory effects of specific *Bothrops* sp. toxins

Among biological active toxins isolated from *Bothrops* sp. venoms, metalloproteases, phospholipases A_2_ and C-type lectin-like proteins play a relevant role by activating platelet function, the coagulation cascade, and the inflammatory host response (50, 103, 104).

#### 4.2.1 *Bothrops* sp. venom SVMPs

The innate immune response may be initiated by a large diversity of *Bothrops* SVMPs. Among them, jararhagin isolated from *Bothrops jararaca (Wied, 1824)*, BaP1 from *Bothrops asper (*Garman, 1883*)*, batroxase from *B. atrox*, neuwiedase from *Bothrops neuwiedi (*Wagler, 1824*)*, moojenactivase from *Bothrops moojeni (*Hoge, 1966*)*, HF3 from *B. jararaca* can activate many features of the immune response, including priming of monocyte/macrophage immune competent cells, neutrophil recruitment and activation, release of proinflammatory cytokines and chemokines, and activation of the complement cascade (C5a and C3a release) (59, 103, 104). For example, jararhagin can elicit the recruitment of inflammatory cells and induce the release of inflammatory mediators such as IL-1β, IL-6, IL-8, and IL-11 *in vitro* (106, 107). Likewise, batroxase can induce the release of pro-inflammatory cytokines (e.g., IL-6, IL-1β, IL-10) and increase the local release of PGE_2_ prostaglandins (108). BaP-1 activates the complement system (release of C5a), induces leukocyte infiltration and mast cells degranulation, as well as cytokine release (109, 110).

In addition to their direct effects on the immune competent cells, SVMPs can indirectly activate platelets by diverse mechanisms, such prothrombin activation, vWF, factor X, and II and the complement cascade (C5a and C3a) release, along with engagement of platelet glycoprotein receptors (103, 104). For example, berythractivase help to upregulate tissue factor (TF) expression in endothelial cells *in vitro*, which favor a systemic thrombogenic and inflammatory activities (111, 112). Likewise, moojenactivase induce factor X, and II activation and platelet tissue factor (TF) expression leading to thrombosis and inflammation (113). SVMPs, such as botrocetin and jararhagin, can engage platelet glycoprotein receptors, which also favor thrombosis and inflammation (114, 115).

#### 4.2.2 *Bothrops* sp. venom phospholipases A2

Several PLA_2_ have been shown to induce a wide range of inflammatory effects (116). Snake venom PLA_2_ also play a role in inflammation, intervening in microvascular permeability, edema formation, leukocyte recruitment and cytokine release (104, 117, 118). Among snake venom PLA_2_, bothropstoxins from *Bothrops jararacussu (*Lacerda, 1884*)* can induce mast cell degranulation and stimulate neutrophil chemotaxis by releasing leukotriene B4 (LTB4) and platelet-activating factor (119). Snake venom PLA_2_ such as batrox PLA_2_ from *B. atrox*, BJ-PLA_2_-I from *B. jararaca* and piratoxin from *Bothrops pirajai* (Amaral, 1923) can also induce mast cell degranulation, stimulate neutrophil recruitment, and increase the production of various cytokines and chemokines (108, 119–122). Snake venom Lys49 PLA_2_ homologs MT-II and MT-III from *B. asper* activate the inflammatory process through NF-kB activation and can increase macrophage phagocytic activity (108). A purified PLA_2_ from the venom of *B. lanceolatus* was recently shown to increase the production of TNF-α, CXCL8, CCL2 and CCL5 and activate the complement system (C5a and C3a release). The venom PLA_2_ also triggered the generation of lipid mediators, as evidenced by the detected high levels of LTB4, PGE2 and thromboxane TXB2 prostanoid (123).

#### 4.2.3 *Bothrops* sp. venom C-type lectin-like toxins

C-type lectin family comprises proteins that bind carbohydrates in a Ca^2+^-dependent manner and non-sugar-binding snake venom C-type lectin-related proteins (SV-CLRPs), so called snaclecs. Snaclecs from snake venom interact with several proteins or receptors having a role in thrombus formation and inflammation, which include C-type lectin-like receptor 2 (CLEC-2), coagulation and vWF factors, GPIb and GPVI receptors on platelets as well as α2β1 receptors of integrins (103, 104). Snaclecs are known to modulate platelet aggregation and their proinflammatory activities. For example, pro-inflammatory activity of *B. jararaca* on mouse and human platelets has been recently described (103, 104). Likewise, engagement of platelet glycoprotein receptors and prothrombin activate platelet can stimulate the pro-inflammatory host response. More specifically, snaclecs from *B. jararacussu* and *Bothrops leucurus* venoms can stimulate immune competent cells (mononuclear cells and neutrophils) to produce proinflammatory mediators (124, 125). Galatrox, a glycan-binding protein from *B. atrox* snake venom promotes neutrophil migration and induces the release of pro-inflammatory cytokines, such as IL-1 and IL-6 both *in vitro* and *in vivo*. Galatrox also stimulates macrophages to produce pro-inflammatory mediators through the TLR4-MyD88 signaling pathway suggesting its role in mediating the proinflammatory action of *B. atrox* venom (126). Likewise, BJcul from *B. jararacussu* venom can activate NLRP3 inflammasome through TLR4 signaling pathway and also induce the activation of NF-κB, resulting in the release of several cytokines such IL-1β and proinflammatory mediators (124–127).

#### 4.2.4 *Bothrops* sp. venom serine proteases

SVSPs are monomeric glycoproteins displaying proteolytic activites that are directly involved in the coagulation machinery by inducing platelet aggregation and activation of coagulation factors. The activity of serine proteases has been mainly correlated to the thrombin-like activity of *Bothrops* sp. venoms, but “kininogenases” present in these venoms have also been shown to participate to inflammatory processes (103, 104). Recent findings suggest the participation of SVSPs in the local and systemic inflammation processes induced by crude *Bothrops* sp. venom. SVSPs from *B. pirajai* snake venom, BpirSP27 and BpirSP41, can promote neutrophil recruitment in the peritoneal inflammatory exudate (108). In contrast, the SVSP batroxobin from *B. moojeni* is a defibrinogenating agent that can inhibit human NETs induced by TNF-α (128). Beside *Bothrops* sp. SVSPs, KnBa from the African viper Bitis arietans can increase the production of IL-1β, TNF α, and IL-6 and also upregulated chemokines such as IL-8, RANTES and MCP-1 (129).

As stated above, the most described activity of SVSPs is thrombin-like, but theses pro-coagulant enzymes also present other activities, such as kallikrein-like, platelet aggregation, and activators of the following substrates: plasminogen, factor X, factor V, prothrombin, and protein C, which may be involved in several inflammatory processes (120). Notably, certain kallikrein-like SVSPs are known to generate vasoactive kinins from α kininogens, which are involved in the regulation of blood pressure, vascular permeability, and inflammatory processes (130). Among others, kininogenase from *B. jararaca*, BjussuSP-I, from *B. jararacussu*, leucurobin from *B. leucurus*, and BpSP-I from *B. pauloensis* venoms display kallikrein-like activity and elicit the release of kallikrein that directly liberates bradykinin and derived vasoactive proinflammatory peptides (131–133). Following B1 and B2 receptor binding, bradykinin can induce numerous pathophysiological processes, including expression of adhesion molecules, leukocyte infiltration and formation of inter-endothelial gaps and protein extravasation (134–136).

### 4.3 Inflammation initiated by whole *Bothrops* sp. venoms

The inflammatory response triggered by *Bothrops* venoms involves platelet activation, leukocyte recruitment (primarily polymorphonuclear and mononuclear cells at the injury site), and the participation of endothelial cells and resident immune cells, which release cytokines in response to the venom (50, 90, 103). In *Bothrops* envenomation, both local and systemic inflammation can occur. A hallmark feature of local tissue damage caused by *Bothrops* venoms is blister formation, characterized by the accumulation of protein-rich fluid due to inflammatory exudation. Analyses of wound exudates and blister fluid consistently reveal elevated levels of pro-inflammatory mediators (41, 45, 137, 138). Additionally, among the numerous DAMPs detected, the most abundant proteins in these exudates are linked to platelet degranulation, innate immune activation, complement pathways, and coagulation cascade.

### 4.4 The role of platelets and neutrophils in inflammation caused by *Bothrops* venoms

In addition to hemostasis, platelets are involved in a multitude of physiological and pathological processes, including the innate immune response induced by *Bothrops* venoms. It is well established that *Bothrops* venoms exert effects on platelets, which are known to be affected by mechanisms including binding or degradation of vWF or platelet receptors, activation of protease-activated receptors (PARs) by thrombin-like enzymes, and modulation of adenosine diphosphate (ADP) and thromboxane A_2_ release (26, 50, 103, 139).

Neutrophils, the primary component of the innate immune system’s initial response, have been identified as a key player in the context of inflammation induced by *Bothrops* venoms (140). Upon activation, neutrophils produce substantial quantities of pro-inflammatory cytokines and are capable of releasing neutrophil extracellular traps (NETs), thereby influencing the course of inflammatory processes (50, 140). The effects of *Bothrops* venoms on neutrophils have been extensively studied, resulting in a substantial body of knowledge accumulated over decades (140).

### 4.5 Activation of complement system, endothelial response and inflammatory mediator release caused by *Bothrops* venoms

*Bothrops* venoms have the capacity to activate the complement cascade, resulting in the generation of substantial quantities of anaphylatoxins, including C3a, C4a, and C5a (108, 141–145). These anaphylatoxins are regarded as the pivotal mediators between innate and adaptive immunity. Another typical characteristic of the systemic inflammatory syndrome induced by *Bothrops* venoms is the cytokine and chemokine storm, which reflects the emergence of multiple disorders in the regulation of the immune response. Once more, a plethora of proinflammatory activities has been observed in *Bothrops* sp (141–145).

*B. lanceolatus* venom exerts a profound impact on the complement system, revealing that the toxins activate both the alternative and classical complement pathways, unbalancing the homeostasis of this immune system (143). Activation of the alternative pathway is accompanied by a paradoxical inhibition of its lytic activity, while the classical pathway is activated by the cleavage of C1 inhibitor by proteases present in the venom. The convergence of the three complement pathways results in the formation of C5 convertase, which cleaves C5 into C5a and C5b. The C5a fragment, a potent anaphylatoxin, induces inflammation and the recruitment of inflammatory cells (146, 147). An elegant study of Delafontaine et al. indicates that metalloproteases in the venom are primarily responsible for the generation of C5a (143). The C5b portion initiates the assembly of the membrane attack complex (MAC), which forms pores in the cell membrane, leading to cell lysis. In addition to this effector function, the complement system, when activated by the venom, performs other biological functions, such as opsonization of pathogens, formation of NETs by neutrophils, and release of anaphylatoxins (C3a and C5a), which amplify the inflammatory response (148–150). On the other hand, venoms of *B. atrox* inhibit the activation of the complement system by the alternative pathway (144). Thus, contribution of the complement system to thromboinflammation in snakebite still remains unclear and requires further exploration.

An *ex vivo* model based on human whole blood demonstrated that *B. lanceolatus* venom elicited an inflammatory reaction comprising the production of pro-inflammatory interleukins (IL-1β, IL-6 and TNF-α), chemokine upregulation (MCP-1, RANTES and IL-8), complement activation and eicosanoid release (leukotriene, prostaglandin and thromboxane) (142). Similarly, the administration of *B. lanceolatus* and *B. atrox* venoms in rats has been observed to result in elevated levels of plasmatic proinflammatory cytokines, including interleukin-1 β (IL-1β), interleukin-6 (IL-6), tumor necrosis factor-alpha (TNF-α), and monocyte chemoattractant protein-1 (MCP-1) (28). In the latter study, plasmatic proinflammatory mediator levels were observed to be higher in rats treated with *B. lanceolatus* compared to those treated with *B. atrox*.

Previous *in vivo* and *in vitro* studies have yielded consistent results regarding the deleterious effects of *Bothrops* venoms on endothelial cell integrity and function (26, 50, 90). Degradation of basement membrane and the subsequent disruption of endothelial cell integrity have been described (151). Detachment of endothelial cells from their surrounding basal lamina, leading to discontinuity of endothelial cell line and extravasation (111, 152, 153). While many SVMPs have no direct cytotoxic effect on capillary endothelium, jararhagin can decrease endothelial cell viability and induce cellular apoptosis, which can be reinforced by phospholipase A_2_ action (111).

An organ‐on‐a‐chip approach used to investigate the effects of various venoms on a perfused microfluidic blood vessel model have recently suggested that *endothelial* barrier function of the microvasculature can be affected by two different mechanisms, including disruption of the endothelial cell membrane and delamination of the endothelial cell monolayer from its matrix (154). SVMPs toxins, isolated from *Bothrops* venoms, induce the expression of adhesion molecules on the microvasculature of murine cremaster muscle. *In vivo*, intravenous injection of *B. jararaca* venom in rabbits induces endothelial injury as evidenced by increase plasma soluble thrombomodulin levels (155). Likewise, SVMPs isolated from venom can render endothelial cells highly thrombogenic, with the release of vWF and expression of tissue factor TF, ICAM-1 and E-selectin (155). SVMPs can also cleaves endothelial glycocalyx proteoglycans, which participle to the disruption of microvasculature integrity (156). In the case of *B. lanceolatus* venom, endothelial injury as evidenced by ICAM-1, VCAM-1, E-selectin, and TF expression, seem to be particularly low compared to *B. jararaca* venom, while longer times of incubation enhanced *B. lanceolatu*s venom induced cytotoxicity (143, 157). Overall, based on these experimental findings, *B. lanceolatus* venom exhibits poor direct endothelial cell toxicity, while intermediate system such as the involvement of the complement system may activate endothelium *in vivo*.

The link between the complement system and thrombosis is complex and multifaceted. Although complement is primarily known for its immunological function, recent studies have demonstrated its involvement in inflammatory processes and blood coagulation. One point that is noteworthy is that C3 is a target of *Bothrops* toxins, and in stroke, elevated plasma C3 levels are markers of worse prognosis in patients with ischemic stroke. In fact, in brain tissue, C3 is produced locally and its activation contributes to ischemic injury (158). Different mechanisms by which the complement cascade can activate coagulation (immunothrombosis) have been reported. Elevated concentrations of complement C3 in the normal population are associated with an increased risk of venous thromboembolism (VTE) (159). When activated, C3 plays a crucial role in amplifying thrombus formation by activating platelets and modulating tissue factor (TF) function by inducing conformational changes in TF, increasing its procoagulant activity and facilitating the exposure of phosphatidylserine (160). C5a can promote the release of prothrombotic factors from platelets, induce the expression of endothelial tissue factor, and promote the natural production of anticoagulants. Other complement components may promote fibrinogen cleavage and increase XIIIa activity, among other mechanisms (161, 162). Therefore, complement dysregulation disorders, such as those caused by *Bothrops* venoms, may result in a prothrombotic state and thrombotic microangiopathy (small vessel thrombosis).

## 5 Immunothrombosis and thromboinflammation

### 5.1 Immunothrombosis

“Immune thrombosis” refers to an excessive inflammatory response that leads to thrombotic events. The concept of “immunothrombosis,” first introduced by Engelmann and Massberg (1), describes a thrombus formation process mediated by immune cells and thrombosis-related molecules. This mechanism aids in pathogen recognition, damaged cell detection, and containment of microbial dissemination in circulation (1, 3–5). Immunothrombosis thus represents an evolutionarily conserved connection between coagulation and innate immunity (163). In contrast, “thromboinflammation” denotes a concurrent inflammatory and thrombotic response occurring in microvessels following exposure to harmful stimuli, such as pathogens or DAMPs (3, 6–9).

Immunothrombosis is emerging as a distinct host defense mechanism against infection, employing specialized molecular pathways to enhance antimicrobial protection. This process enables pathogen recognition, limits microbial dissemination, and contributes to vascular immunity by integrating coagulation and immune responses within the bloodstream (1, 3–5). Microbial components, designated as pathogen-associated molecular patterns (PAMPs), are recognized by pattern recognition receptors (PRRs) on immune competent cells, such as monocytes (164). Following the recognition of the pathogen, monocytes present activated tissue factor (TF) on their surfaces, which is released *in situ*, thus activating the extrinsic pathway of coagulation (165, 166). Monocytes release decrypted TF in a process called pyroptosis, which provokes leakage in response to NLRP3 inflammasome and caspase pathway (IL-1β and IL-18) activation (4). Additionally, monocyte activation can result in the release of pro-inflammatory cytokines (165, 166). Proinflammatory molecules recruit neutrophils, which contribute to immunothrombosis through the release of NETs. NETs directly activate factor XII, thereby initiating the contact-dependent pathway of coagulation. NETs bind von Willebrand factor (vWF) and facilitate the recruitment and activation of platelets. NETs cleave and inactivate natural anticoagulants, including tissue factor pathway inhibitor and thrombomodulin (166–170). Additionally, NETs can externalize and bind tissue factor TF, which further promotes the activation of the extrinsic pathway of coagulation (171).

Immunothrombosis describes an overshooting inflammatory reaction that results in detrimental thrombotic activity. The major pathological outcome is thrombosis (occlusion of a blood vessel) due to platelet-involved thrombotic activity in response to initial inflammatory stimuli, such as pathogen invasion. Upon activation, platelets promote the immunothrombotic process by triggering the contact-dependent pathway of coagulation through the release of polyphosphates. In collaboration with endothelial cells, they facilitate the generation of fibrin (172, 173). Once activated, platelets release substantial quantities of pro-inflammatory cytokines, thereby contributing to the establishment of an inflammatory microenvironment. As a result of this mechanism, pathogens are trapped within the fibrin-based NETs and eliminated in this intravascular restricted compartment (3–5, 7).

In this scenario, immunothrombosis is primarily initiated by monocytes and neutrophils and is facilitated by the formation of microthrombi in microvessels (167, 168, 174–176). It is of particular importance to note the crucial role played by tissue factor, monocyte triggering via NLRP3 inflammasome activation, release of NETs, and activated platelets in the cross talk of inflammation with the coagulation processes. It is noteworthy that the term “immunothrombosis” is now employed in a more expansive manner to encompass thrombotic events driven by infection or sterile inflammation. The primary consequence of immunothrombosis is a process of microcoagulation, which does not elicit an adverse systemic response and effectively immobilizes invading pathogens or foreign “alarmin” structures (in the context of sterile inflammation) for subsequent clearance by immune competent cells (1, 3–5).

### 5.2 Thromboinflammation

This term denotes a process whereby inflammation and thrombosis coexist within microvessels in response to noxious stimuli, including pathogens, injured cells, and other harmful molecules. Thromboinflammation represents the manifestation of dysregulation of the two most crucial defensive and wound-healing responses of the body: inflammation and hemostasis (3, 6–9). Thromboinflammation describes the interplay of platelets and coagulation with the vascular system, resulting in the recruitment of immune cells. In this process, the initial platelet adhesion/activation pathways act in concert with key components of immune cells and the contact pathway of plasmatic coagulation (factor XII—kallikrein/kinin pathway) (5, 12, 166, 172). Intravenous thrombotic processes can, in turn, trigger aberrant complement, coagulation, platelet, and endothelial cell activation, which may ultimately result in disrupted vascular integrity. It is well-established that viral and bacterial infections, as well as ischemia–reperfusion (e.g., acute ischemic stroke, coronary heart disease), can cause microvascular thrombi and fuel inflammatory processes (3, 6–11, 13). Overall, thrombo-inflammation describes the interplay of platelets and coagulation with the immunovascular system. The major pathological outcome is resulting in the recruitment of immune cells.

## 6 Thromboinflammation induced by *Bothrops* venoms

Thromboinflammation triggered by *Bothrops* snakebite envenoming represents a distinct pathophysiological pathway with unique biological characteristics (3, 6–9). **Supplementary Table 2** summarized the main characteristics of thromboinflammation in *Bothrops* sp. envenoming comparing the effects of venoms with hemorrhagic profile with those with thrombotic profile.

Previous research has suggested an interaction between inflammatory and coagulation processes in these envenomation cases (50). A recent investigation of *B. atrox* bite victims demonstrated that fibrinogen concentrations modulate inflammatory mediator responses (177). The researchers found that fibrinogen levels directly influence cytokine and chemokine expression patterns including CXCL-8 CXCL-9 CCL-2 and IL-6. They also documented elevated CCL-5 levels alongside decreased IFN-γ concentrations in patients with reduced plasma fibrinogen. This study provided the first evidence that thromboinflammation involving reciprocal interactions between inflammation and coagulation mechanisms occurs in *Bothrops* envenomation cases (177).

### 6.1 Role of platelets

Platelets mediate a harmful interaction between coagulation pathways and immune cells. Within the framework of thromboinflammation this dysregulated crosstalk leads to NETs formation and activation of immune-competent cells including monocytes (178). The P-selectin/PSGL-1 axis serves as a crucial mediator of cellular interactions involving endothelial cells immune cells and neutrophils. P-selectin remains stored within α-granules of resting platelets and Weibel-Palade bodies of endothelial cells. PSGL-1 functions as the principal receptor for P-selectin facilitating neutrophil recruitment and fostering a proinflammatory milieu through monocyte/macrophage activation (3 6 173). Extensive research has characterized the impact of *Bothrops* venoms on platelet aggregation and activation (179–181). However relatively few studies have examined adhesion molecule expression on platelet surfaces following *Bothrops* venom exposure. Both *in vitro* and *in vivo* experiments have demonstrated that *Bothrops* venoms can upregulate various adhesion molecules including L-selectin integrin αLβ2 (LFA-1) ICAM-1 PECAM-1 and CD18. Notably only a single investigation has reported P-selectin expression on platelet surfaces during the early hours following *B. jararaca* envenomation in rabbits (182). These findings underscore the need for greater emphasis on understanding the P-selectin/PSGL-1 pathway’s role in *Bothrops* envenomation pathophysiology.

### 6.2 Role of leukocytes

Although the interaction between platelets and monocytes/leukocytes through P-selectin-PSGL1 binding has not been specifically investigated, the increased expression of E-selectin and L-selectin induced by *Bothrops* venoms has been demonstrated to facilitate leukocyte rolling and adhesion to the endothelium (28, 183–185). In accordance with the aforementioned findings, an elegant study employing intravital microscopy has demonstrated that jararhagin, a multi-domain snake venom metalloproteinase isolated from *B. jararaca* venom, can increase the number of rolling leukocytes in post-capillary venules of mouse cremaster muscle (186). Furthermore, *in vivo* studies have demonstrated that *Bothrops* venoms can induce the migration of polymorphonuclear neutrophils to the envenoming sites and function as phagocytes and inflammatory response controllers (141, 184–186).

As part of the host defense mechanisms against snake venom reaction, neutrophils generate reactive oxygen species (ROS), produce several proinflammatory cytokines and eicosanoids, and release of NETs (140). NETs production represents a convergence point for the processes of inflammation, coagulation, and thrombosis (3–5, 12, 168, 169, 171, 176, 187, 188). NETs are regarded as a crucial element in the thrombotic process, as they intensify platelet and endothelial cell activation and facilitate fibrin formation in *Bothrops* envenoming (87, 88). *In vitro* and *in vivo* studies have demonstrated that *Bothrops* venom can induce NET formation (131, 189–191). Several toxins of snake venoms were able to induce *in vitro* DNA release from human neutrophils. For example, BaTX-II, a phospholipase A_2_ from *B. atrox* induced the release of double strand DNA from neutrophils collected from healthy donors (190). Similar results have been obtained with BjussuMP-II, a P–I class of SVMPs from the *B. jararacussu* (191).

### 6.3 Role of high molecular weight vWF multimers

The vascular occlusion observed in thromboinflammation depends critically on interactions between ultra-large von Willebrand factor (vWF) and NETs (2, 9, 167, 174–176). Research has shown that *Bothrops* snake venoms can directly influence vWF polymerization in circulating blood (189). Experimental studies in rats revealed temporary reductions in plasma ADAMTS13 concentrations following envenomation by *B. jararaca* and *B. lanceolatus* (28, 192). Interestingly these venom-induced decreases in ADAMTS13 activity did not consistently lead to elevated vWF antigen levels or a shift toward ultra large and high molecular weight vWF multimers in circulation. This apparent paradox has been explained by the proteolytic degradation of these multimers through the action of venom metalloproteinases (192–194).

The lack of detectable ultralarge vWF multimers in plasma does not exclude their potential role in thromboinflammation. These multimers may still participate in NET formation and vascular adhesion particularly when considering venom-induced endothelial activation. The combined effects of immune cell activation endothelial stimulation and impaired vWF cleavage due to reduced ADAMTS13 activity likely promote platelet-vessel wall interactions that drive microthrombus formation. Histological examinations of thrombi from both animal models and human cases consistently demonstrate colocalization of fibrin NETs and vWF within the thrombus structure (2). Supporting these findings microthrombi have been identified in pulmonary vessels of envenomed mice and in postmortem analysis of a case of *B. lanceolatus* envenomation (23, 29).

### 6.4 Thromboinflammation as a unifying mechanism of Bothrops venom-induced thrombosis

To gain a deeper understanding into underlying mechanisms of thrombotic complications in *Bothrops* snakebite envenoming, it is essential to consider two lines of evidence. Firstly, the absence of overt consumption coagulopathy due to the weak procoagulant effects of the venoms of these snakes may be associated with the thrombotic effects, as clotting factors are present in the bloodstream by the time the thrombogenic and inflammatory mechanisms induced by the venom are operating in blood vessels (28). Secondly, in addition to the presence of normal coagulation factors and a proinflammatory response, it is necessary to examine whether the biological response elicited by *Bothrops* venoms exhibits the typical signature of thrombo-inflammation.

**Figure 1:**
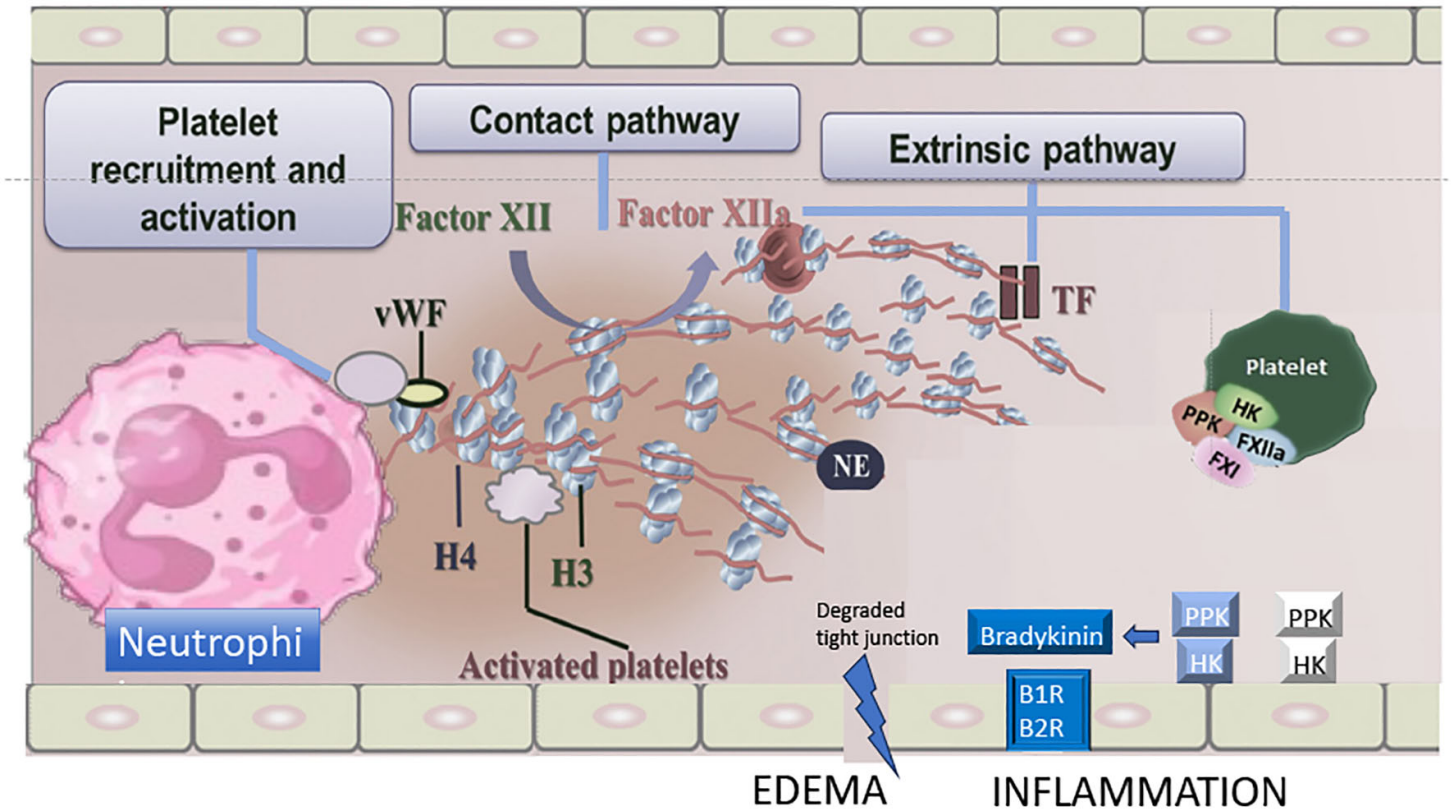
Major drivers involved in inflammation and coagulation activation eventually leading to thromboinflammatory pathways in *Bothrops* sp. envenoming. Proposed mechanisms of contact phase activation with NET formation, extrinsic coagulation, von Willebrand factor (vWF) and kallikrein/bradykinin pathway following *Bothrops* sp. venom exposure. H, histone; NE, neutrophil elastase; TF, tissue factor; HK, high-molecular-weight kininogen; PPK, plasma prekallikrein; B1R/B2R, bradykinin 1/2 receptors. *Adapted from Teixeira C* et al. *Inflammation Induced by Platelet-Activating Viperid Snake Venoms: Perspectives on Thromboinflammation. Front Immunol. 2019;10:2082. doi:10.3389/fimmu.2019.02082*.

*B. lanceolatus* and *B. caribbaeus* venoms, that display a prothrombotic profile, can exhibit specific biological characteristics that are identical to those observed in thromboinflammation. Firstly, it can be argued that the lack of overt consumption coagulopathy due to the weak procoagulant effects of *B. lanceolatus* and *B. caribbaeus* venoms in comparison to other *Bothrops* venoms is a crucial factor that allows for a hypercoagulable state. Similarly, it has been demonstrated that the systemic proinflammatory response elicited by *B. lanceolatus* venom is more pronounced than that induced by *B. atrox* venom (28). Therefore, the thrombotic effects of *B. lanceolatus* venom may be attributable to the combined action of clotting factor activation and the concurrent operation of potent inflammatory mechanisms within the blood vessels (123, 142, 143, 157). Secondly, an increasing body of evidence indicates that *B. lanceolatus* and *B. caribbaeus* venoms exhibit distinctive characteristics of thromboinflammation, which are not observed in other *Bothrops* venoms. For example, distinct kallikrein-like activity and ADAMTS13/von Willebrand factor (vWF) interactions have been observed in *B. lanceolatus* and *B. atrox* venoms (30). A summary of major drivers involved in inflammation and coagulation activation eventually leading to thromboinflammatory pathways in *Bothrops* snakebite envenoming is displayed **Figure 1**.

Before concluding that *Bothrops* sp. envenoming is related to thromboinflammation, it is important to consider the dualistic effects of snake venoms on hemostasis. Snake venoms are intricate biochemical arsenals that often contain toxins with opposing effects on hemostasis, including both procoagulant and anticoagulant factors, as well as platelet-activating and platelet-inhibiting components (16, 26). The resulting disturbances in hemostasis and inflammation can arise through independent, synergistic, or antagonistic mechanisms, contributing to the diverse and sometimes paradoxical effects observed in snakebite victims. At first glance, anticoagulant toxins and thromboinflammation appear to represent opposing mechanisms—anticoagulants prevent clotting, while thromboinflammation involves thrombosis-driven inflammatory responses. Nevertheless, despite their anticoagulant effects, some venom toxins can indirectly drive thromboinflammation through the cleavage of fibrinogen that releases fibrinopeptides and other breakdown products, which may activate TLR4, elicit pro-inflammatory cytokine release and leukocyte recruitment (104). Likewise, some anticoagulant toxins (e.g., disintegrins) block platelet aggregation, but can activate platelets (179–181), which may release microparticles (procoagulant surfaces) and serotonin, along with P-selectin expression and leukocyte recruitment creating a prothrombotic microenvironment and vascular inflammation. Overall, antiplatelet toxins do not simply prevent clotting—they shift thrombosis to alternative pathways (platelet activation, NETosis, tissue factor-driven coagulation, endothelial injury).

## 7 Advances and perspectives for the study of thromboinflammation in snakebite envenoming

The interaction between hemostasis and activation of innate immunity is highly complex, which makes it difficult to precisely define the relative contribution of each of these two processes to the pathogenesis of different complications, possibly explaining the absence of a direct association between classical biomarkers of hemostasis activation and the risk or severity of some of these clinical manifestations. Thus, the crossover between inflammation and thrombosis is very well exemplified during snakebite envenoming, due to the presence of a wide variety of characterized proteins that can activate the innate immune system and/or hemostasis.

Evidence supports the crossover between inflammation and hemostasis: (i) studies in animal models report hemostasis disorders; despite some heterogeneity within the model and within the venom, a less effective hemostatic system is associated with an increase in hemorrhagic manifestations; (ii) it has been shown that components of the coagulation system, such as platelets, also signal through immunological pathways; (iii) there are examples of toxins and venoms whose mechanisms disrupt local hemostatic balance and induce inflammation; and (iv) studies show that leukocytes are not only found at the site of envenoming, but also in arterial and venous thrombi. However, it is noteworthy that to date, little studies about *Bothrops* snakebite envenoming causing thrombotic complications have been carried out.

To advance our understanding of the role of thromboinflammation in the development of thrombotic clinical complications in *Bothrops* snakebite envenoming, it is necessary to develop new study models and apply advanced study techniques. This limitation to date is due to the use of less robust techniques and studies that report changes in only one of the axes of thromboinflammation, resulting in incomplete reports and fragmented conclusions. It is important to mention that research on thromboinflammation involves a variety of approaches, and some are proteomics, animal models, biomarkers for thromboinflammation measure, use of image techniques and others. This will not be an easy task for the scientific community, and among the challenges we can list the difficulty associated with (i) multiple molecular interactions, considering that thromboinflammation involves a complex cascade of biochemical reactions, with the participation of several cells and molecules; (ii) heterogeneity of the disease, since the clinical manifestations of thromboinflammation vary widely between different diseases and individuals, making it difficult to create universal models, especially for snake envenoming; (iii) study of the inflammatory microenvironment, which is dynamic and heterogeneous, influencing the progression of thrombosis.

Proteomics is a useful tool for studying thromboinflammation in a more comprehensive manner, allowing us to identify changes in biochemical processes related to hemostasis and inflammation (complement system, inflammatory cells, cytokines, chemokines) and other elements to thromboinflammation (195–197). This can be done using animal models, which is a limiting factor, because other models have already been developed to study the pulmonary thrombotic effect induced by *Bothrops* snake venom (29), we do not yet have models for cerebral thrombotic complications. Studies characterizing biomarkers of thrombo-inflammation in patients with cerebral thrombotic complications from *Bothrops* snakebite envenoming will also be useful, as will the use of proteomics to study these cases (198). The use of imaging techniques, such as computed tomography and magnetic resonance imaging, can be used to visualize thrombus formation and assess the impact of thromboinflammation on different organs. The use of organoids and organ chips will allow for more realistic simulation of the *in vivo* microenvironment, genetically modified animal models to study the role of specific pathways and proteins in thromboinflammation, the analysis of large data sets and the application of machine learning algorithms; and finally, collaboration between different areas, such as biology, medicine, engineering, and computer science, will be essential to overcome the challenges of modeling thromboinflammation.

## 8 Conclusions

*Bothrops* snake venom, which is common in tropical regions, has a complex composition of toxins that can trigger a series of systemic effects, including coagulopathies and thrombotic events. Understanding the exact mechanisms involved in this pathogenesis is still a challenge for the scientific community, but some theories have been proposed to explain this complex interaction between the venom and the human organism. The thromboinflammation theory has emerged as one of the main hypotheses to explain the effects of *Bothrops* venom.

Thrombotic complications are extremely rare in *Bothrops* snakebite envenoming. Several factors can contribute to the development of ischemic stroke in patients bitten by *Bothrops* snakes, which include the procoagulant activity of venom toxins, hypovolemic shock, and endothelial dysfunction and injury. In the specific cases of *B. lanceolatus* and *B. caribbaeus*, thrombotic events are frequent. Proposed mechanism has been related to the combination of two processes: a weak procoagulant effect of *B. lanceolatus* and *B. caribbaeus* venoms, which does not induce coagulation factor consumption, along with the simultaneous activation of thrombogenic and inflammatory processes operating in blood vessels. Despite early and adequate initiation of treatment for *B. lanceolatus* and *B. caribbaeus* envenoming, the patient can develop a catastrophic stroke, resulting in significant disability. Proposal of thromboinflammation as a key pathophysiological event in these envenoming will provide options for the development of new therapeutical issues, which may target antithrombin, activated protein C, thrombomodulin, glycosaminoglycans and interleukin inhibitors.
