# Supplementary material for: Thromboinflammatory complications of Bothrops snakebite envenoming: the case of B. lanceolatus endemic to the Caribbean Island of Martinique
Source: Front Immunol. 2025 Sep 10;16:1625165. doi: 10.3389/fimmu.2025.1625165 (PMC12457118; doi:10.3389/fimmu.2025.1625165)
Supplement: Supplementary file 1 [file Table1.docx]

**Supplemental Table 1**. Thrombotic complications in *Bothrops* snakebite envenoming

| **Reference**  **First author date**  **Ref. number** | **Study**  **Period and location** | **Snake** | **Patients** | **Treatment** | **Clinical**  **complications** | **Mortality** | **Platelets** |
| --- | --- | --- | --- | --- | --- | --- | --- |
| Thomas 1994  (1) | 1986-1992  Martinique | *Bothrops lanceolatus* | 64 | none | myocardial infarction (5 cases), pulmonary embolus  (1 case), cerebral infarction (7 cases), femoral artery thrombosis  (1 case), combined myocardial and cerebral infarction (2 cases), combined myocardial infarction and pulmonary embolus (1 case) | 8 deaths | < 100 000/mm^3^ in 15 patients |
| Thomas 1995 and Thomas 1996  (2,3) | 1991-1995  Martinique | *Bothrops lanceolatus* | 72 | antivenom (AVS) in 45 patients | pulmonary embolism (2 cases), cerebral infarction (6 cases), myocardial  infarction (1 case), and combined cerebral and myocardial infarctions (2 cases) in untreated AVS patients | 3 deaths in untreated AVS patients;  none in AVS treated patients | < 100 000/mm^3^ in 6 patients among 11 who developed thrombosis |
| Bucher 1997  (4) | 1993-1995  Martinique | *Bothrops lanceolatus* | 40 | 32 patients received AVS | none | none | Normal range |
| Numeric 2002  (5) | 2002  Saint Lucia | *Bothrops caribbaeus* | Single case report |  | multiple areas of cerebral ischemia, especially in the right anterior cerebral artery territory | none | 201 000/mm^3^ |
| Angarita Diaz 2003  (6) | 2003  Colombia | *Bothrops sp* | Single case report | AVS | cytotoxic /vasogenic edema in the right middle cerebral artery | none | < 50 000/mm^3^ |
| Mosquera  2003  (7) | 2000-2001  Ecuador | *Bothrops sp* | Single case report | NA | ischemic stroke (1 case)  in the territory of the left middle cerebral artery, the right posterior cerebral artery, andboth superior cerebellar arteries | NA | NA |
| Merle 2005  (8) | 2005  Martinique | *Bothrops lanceolatus* | Single case report | AVS | right occipital infarction | none | 52 000/mm^3^ |
| Thomas 2006  (9) | 2006  Martinique | *Bothrops lanceolatus* | Three case reports | AVS | acute bilateral occipital infarcts; acute ischemic stroke; acute bilateral small hemispheric cortical infarcts | none | < 100 000/mm^3^ in 2 patients |
| Malbranque 2008  (10) | 2008  Martinique | *Bothrops lanceolatus* | Single case report | AVS | Combined multiple cerebral infarcts  typical junctional distribution and myocardial  infarction | deceased | < 100 000/mm^3^ |
| Canas 2016  (11) | 2016  Colombia | *Bothrops atrox* | Single case report | AVS- | ischemia in the brainstem | none | Normal range |
| Florentin  2024  (12) | 2020, 2021  Martinique | *Bothrops lanceolatus* | Two case report | AVS | Case 1: bilateral hemispheric and cerebellar infarcts  Case 2: combined pulmonary embolism and multiple strokes, involving the PICA and junctional territories | none | 2050 00/mm^3^  105 000/mm^3^ |
| Martínez-Villota 2022  (13) | 2021  Colombia | *Bothrops sp.* | Single case report | AVS | multiple ischemic areas in the bilateral frontal-temporal and bilateral occipital regions, as well as pons and cerebellum | none | Thrombocytopenia |
| Galan 2023  (14) | 2023  Brazil | *Bothrops jararaca* | Single case report | AVS | mesenteric ischemia | none | 112 000/mm^3^ |
| Bentes 2024  (15) | 2024  Brazil | *Bothrops sp.* | Single case report | AVS | ischemic cerebral edema and a lesion in the left parietal lobe | none |  |

AVS: antivenom therapy; NA: data non available

**References of Table 1**

1. Thomas L, Tyburn B, Ketterle J, Rieux D, Garnier D, Smadja D. Coagulopathy and thrombosis in human victims of bothrops lanceolatus envenoming in Martinique. *Réanimation Urgences* (1994) 3: 25-30. doi:10.1016/S1164-6756(05)80309-3
2. Thomas L, Tyburn B, Bucher B, Pecout F, Ketterle J, Rieux D, Smadja D, Garnier D, Plumelle Y. Prevention of Thromboses in Human Patients with *Bothrops lanceolatus* Envenoming in Martinique: Failure of Anticoagulants and Efficacy of a Monospecific Antivenom. Research Group on Snake Bites in Martinique. *Am J Trop Med Hyg* 1995, *52*, 419–426, doi:10.4269/ajtmh.1995.52.419.
3. Thomas L, Tyburn B, Lang J, Ketterle J. Early Infusion of a Purified Monospecific F(Ab’)2 Antivenom Serum for *Bothrops lanceolatus* Bites in Martinique. *Lancet* 1996, *347*, 406, doi:10.1016/s0140-6736(96)90590-5.
4. Bucher B, Canonge D, Thomas L, Tyburn B, Robbe-Vincent A, Choumet V, Bon C, Ketterlé J, Lang J. Clinical indicators of envenoming and serum levels of venom antigens in patients bitten by Bothrops lanceolatus in Martinique. Research Group on Snake Bites in Martinique. Trans R Soc Trop Med Hyg. 1997 91(2):186-90. doi: 10.1016/s0035-9203(97)90219-4.
5. Numeric P, Moravie V, Didier M, Chatot-Henry D, Cirille S, Bucher B, Thomas L. Multiple Cerebral Infarctions Following a Snakebite by *Bothrops caribbaeus*. *Am J Trop Med Hyg* 2002, *67*, 287–288, doi:10.4269/ajtmh.2002.67.287.
6. Angarita JA, Cárdenas LF. Infarto cerebral y accidente ofídico. *Acta Neurol Colomb* 2003, 19, 75-79.
7. Mosquera A, Idrovo LA, Tafur A, Del Brutto OH. Stroke following Bothrops spp. snakebite. *Neurology* 2003, ;60(10):1577-80. doi: 10.1212/01.wnl.0000061614.52580.a1.
8. Merle H, Donnio A, Ayeboua L, Plumelle Y, Smadja D, Thomas L. Occipital infarction revealed by quadranopsia following snakebite by Bothrops lanceolatus. Am J Trap Med Hyg. 2005;73:583–585.
9. Thomas L, Chausson N, Uzan J, Kaidomar S, Vignes R, Plumelle Y, Bucher B, Smadja, D. Thrombotic Stroke Following Snake Bites by the “Fer-de-Lance”*Bothrops lanceolatus* in Martinique despite Antivenom Treatment: A Report of Three Recent Cases. *Toxicon* 2006, *48*, 23–28, doi:10.1016/j.toxicon.2006.04.007.
10. Malbranque S, Piercecchi-Marti MD, Thomas L, Barbey C, Courcier D, Bucher B, Ridarch A, Smadja, D, Warrell DA. Fatal Diffuse Thrombotic Microangiopathy after a Bite by the “Fer-de-Lance” Pit Viper (Bothrops lanceolatus) of Martinique. *Am J Trop Med Hyg* 2008, *78*, 856–861.
11. Cañas CA. Brainstem ischemic stroke after to Bothrops atrox snakebite. Toxicon. 2016 Sep 15;120:124-7. doi: 10.1016/j.toxicon.2016.08.005.
12. Florentin, J, Farid, K, Kallel, H, Neviere, R, Resiere, D. Case Report: Acute myocarditis and cerebral infarction following Bothrops lanceolatus envenomation in Martinique: a case series. *Front Cardiovasc Med* 2024, 11, 1421911. doi: 10.3389/fcvm.2024.1421911.
13. Martínez-Villota VA, Mera-Martínez PF, Portillo-Miño JD. Massive acute ischemic stroke after Bothrops spp. envenomation in southwestern Colombia: Case report and literature review. Biomedica. 2022 Mar 1;42(1):9-17. doi: 10.7705/biomedica.6114.
14. Galan LEB, Silva VS, Silva VS, Monte RC, Jati S.R, et al. Acute mesenteric ischemia following lancehead snakebite: an unusual case report in the Northernmost Brazilian Amazon. *Front Med* (Lausanne). 2023, 10, 1197446. doi: 10.3389/fmed.2023.1197446.
15. Bentes KO, de Amorim RLO, Barbosa FBA, Ratis da Silva VCP, Valente J, Almeida-Val F, et al. Long-term disability after cerebral ischemic stroke following a Bothrops atrox snakebite in the Brazilian Amazon. *Toxicon* 2024, 247, 107793. doi: 10.1016/j.toxicon.2024.107793.
