# Supplementary material for: Thromboinflammatory complications of Bothrops snakebite envenoming: the case of B. lanceolatus endemic to the Caribbean Island of Martinique
Source: Front Immunol. 2025 Sep 10;16:1625165. doi: 10.3389/fimmu.2025.1625165 (PMC12457118; doi:10.3389/fimmu.2025.1625165)
Supplement: Supplementary file 2 [file Table2.docx]

**Supplemental Table 2**. Key steps of thromboinflammation in *Bothrops* snake displaying hemorrhagic and ischemic clinical features

| **Key steps of thromboinflammation** | | | **Hemorrhagic *Bothrops*** | | **Ischemic *Bothrops*** |
| --- | --- | --- | --- | --- | --- |
| **Steps** | **Thromboinflammation** | ***B. atrox*** | | ***B. jararaca*** | ***B. lanceolatus*** |
| **Recognition of the danger signal “alarmins” and release of inflammatory mediators** | Following inflammation mediated by the NF-κB, MAPK and JAK-STAT, intervention and detection of mast cells and macrophages via their PRR receptors, MAMP or DAMP, and release of chemokines (anaphylatoxins, IL-8), cytokines (IL-6, IL-1ß, IFN-I, TNF-α, etc.), vasoactive amines (serotonin and bradykinin), chemokines (CXCL8, CCL2,3,4, and 5), and lipid mediators (PAF, leukotrienes, prostaglandins, and thromboxane A2)^1^. | Release of IL-6, IL-1β, IL-10, and increased local release of prostaglandins by *B. atrox* batroxase^2^.  Release of IL-1 and IL-6, in vitro and in vivo, by Galatrox, a glycan-binding protein from the venom of *B. atrox*. Galatrox also stimulates macrophages to produce pro-inflammatory mediators via the TLR4-MyD883 signaling pathway^3^. | | Presence of high concentrations of IL-6, IL-8, TNF-α, MIP-1α, and CCL5. Production and release of COX1- and COX2-derived prostaglandins, including PGE2, PGD2, and TXA2, and 5-LO-derived LTB4, due to the action of *B. jararaca* venom on neutrophils and macrophages. Nuclear translocation of NF-κB in human monocytes and mouse alveolar macrophages by *B. jararaca* venom, promoting IL-1β production. Amplification of NF-κB activation by venom through prostaglandin and inhibition of this activation by LTB4^4^.  Recruitment of inflammatory cells and release of IL-1β, TNF-α, IL-6, IL-10, IL-8, and IL-11 *in vitro* by jararhagin metalloproteinase isolated from *B. jararaca*. ^5,6^. | Inflammatory response by the venom of *B. lanceolatus* involving the production of IL-1β, IL-6, and TNF-α, the upregulation of chemokines (MCP-1, CCL5, and IL-8), complement activation, and the release of leukotriene, prostaglandin, and thromboxane through the action of cyclooxygenase and 5-lipoxygenase. Conversion of lysophospholipids into platelet-activating factor (PAF)^7^. Potentiation of TNF-α, CXCL8, CCL2, CCL5 production and generation of lipid mediators such as LTB4, prostaglandins, and TXB2 by purified PLA2 from *B. lanceolatus* venom^8^ . |
| **Platelet adhesion and activation** | Activation of circulating platelets upon contact with subendothelial collagen. Binding of collagen to von Willebrand factor (VWF), which unfolds. Adhesion of platelets to VWF via the GP Ib-IX-V receptor complex or to collagen via the GPVI receptor. Release of inflammatory mediators promoting platelet adhesion, activation, and aggregation, leading to the surface of platelets, binding of TXA2, ADP, and FIIa to G protein-coupled receptors. Then, formation of alpha granules, release of dense granules, and activation of GPIIb/IIIa.^9^ | Low correlation of platelets with tissue factor, factors II and V, D-dimer, plasminogen activity, and moderate correlation with fibrinogen and fibrinogen degradation products (or fibrin). Very low platelet count in patients with systemic hemorrhage^10^.  Activation of platelet PARs by *B. atrox* venom through their thrombin-like enzymes ^4^. Activation of platelet aggregation by thrombocytin, SVSP from *B. atrox*, through a mechanism similar to that of thrombin by hydrolyzing PAR1 and PAR4, which are found on human platelets ^11^. | | Platelet adhesion and aggregation result from the activation of specific receptors. Botrocetin, an SVMP found in the venom of *B. jararaca*, promotes platelet aggregation via the interaction between vWF and GPIb. It first acts by binding to vWF, then this complex interacts with GPIb to induce agglutination. Other SVMPs, such as jararhagin, jaracetine, and a single-chain form of botracetin, can also activate vWF and its binding to GPIb-IX-V. In addition, jararhagin and jaracetine block collagen adhesion to integrin α2β1, unlike botracetin^4^. The venom of *B. jararaca* also activates platelet PARs, leading to platelet aggregation via thrombin-like enzymes and prothrombin activators. It also participates in the release of PF4 and β-hexosaminidase from α granules and platelet lysosomes. Thrombocytin, SVSP from *B. atrox*, activates platelet aggregation through a mechanism similar to that of thrombin by hydrolyzing PAR1 and PAR4, which are found on human platelets. PA-BJ, SVSP from *B. jararaca* activate platelet aggregation through a mechanism similar to that of thrombin by hydrolyzing PAR1 and PAR4, which are found on human platelets, mobilizing calcium in the platelets.^11^. | According to a study, thrombocytopenia caused by *B. lanceolatus* venom observed in mice and no direct action on platelet aggregation in human platelet-rich plasma ^12^. |
| **Involvement of the vWF/ADAMTS13 axis in thromboinflammation** | The thromboinflammatory response is strongly linked to the release of von Willebrand factor (vWF). During inflammation, the activation of endothelial cells exposes vWF, which accumulates in the subendothelial matrix and promotes platelet adhesion and activation as well as the formation of ultra-large vWF. UL-vWF is cleaved by ADAMTS13 into smaller, less reactive multimers of vWF, thereby establishing the vWF/ADAMTS13 axis. The imbalance in the VWF/ADAMTS13 axis, defined by increased VWF levels and decreased ADAMTS13 levels, promotes the thromboinflammatory response by causing hypercoagulation, inhibition of fibrinolysis, neutrophil activation, and interaction with NET DNA.^13^ | Null | | Temporary decreases in plasma ADAMTS13 concentrations without necessarily leading to elevated levels of vWF antigen or a shift to ultra-large, high-molecular-weight vWF multimers in circulation^14–16^. | |
| **Vascular effects** | The secretion of inflammatory mediators will also increase the permeability of the endothelium and dilate blood vessels, facilitating the passage of complement factors (leading to amplification of the inflammatory response via C3a and C5a) and fibrinogen converted to fibrin (thanks to platelet polyphosphates that activate the intrinsic pathway leading to FIIa activation, thrombus formation, and stabilization^17^) which prevents the spread of the pathogen. The mediators also enable the recruitment and adhesion of neutrophils (if effective) or monocytes to endothelial cells, which will migrate to the site of inflammation by diapedesis. In the case of monocytes, once they reach the tissue, they differentiate into macrophages and then lymphocytes^18^. | The procoagulant toxins in *B. atrox* venom activate several coagulation factors (II, X, V, VIII, XIII, and kallikrein^19^) and promote intravascular thrombin generation. The venom also contains fibrinolytic components that degrade fibrinogen and contribute to coagulopathies.^10^.  The venom of *B. atrox* can also degrade fibrin. Galatrox from *B. atrox* promotes neutrophil migration. The venom of B. atrox induces a marked Th1 immune response, characterized by severe local inflammation with high levels of IL-1β, IL-6, TNF-α, CXCL-1, and CXCL-2. This Th1 response is regulated by neutrophils and the MyD88 pathway^10^. According to one study, in cases of envenomation by *B. atrox*, fibrinogen concentration levels influence cytokine (IL-6) and chemokine (CXCL-8, CXCL-9, CCL-2) responses, with patients with low plasma fibrinogen levels also having elevated CCL-5 levels and decreased IFN-γ concentrations^20^. | | Induction of neutrophils to the site of envenomation by venom. *B. jararaca* venom activates platelets, causing their immobilization and the development of thrombin*. B. jararaca* venom can inhibit platelet aggregation induced by ristocetin and collagen^10^.  The venom of *B. jararaca* may have fibrinolytic activity and degrade fibrin. The kininogenase of *B. jararaca* has an action similar to that of kallikrein and induces the release of the latter, which directly leads to the formation of bradykinin and pro-inflammatory vasoactive peptides derived from it.  Involvement of ICAM-1, LECAM-1, LFA-1, and PECAM-1 in neutrophil recruitment by *B. jararaca*^21^. | The venom of *B. lanceolatus* is capable of activating FIIa and kallikrein (intrinsic pathway activation). Results obtained from purified proteins show that the venom of *B. lanceolatus* directly activates prothrombin but is unable to directly activate FX. It appears to have no effect on FX. The activation of FII and kallikrein by *B. lanceolatus* venom highlights its role in fibrin formation, even though it does not affect FXa. *B. lanceolatus* venom can induce fibrin formation in plasma and purified human fibrinogen, indicating activity similar to that of thrombin, as well as fibrinogen degradation.^19^.  Induction of neutrophils to the site of envenomation by venom.^10^ |
| **Activation of the complement system during thrombosis** | During thrombosis, activation of the complement system in conjunction with platelets induces the cleavage of C3 into C3a and C3b and C5 into C5a and C5b. C3a and C5a then potentiate platelet activation and aggregation by inducing the exposure of P-selectin for the recruitment of neutrophils in the endothelium. C5a induces upregulation of TF and expression of plasminogen activator inhibitor-1 on neutrophils, monocytes, and endothelial cells. C5b also contributes to the formation of MAC, which initiates coagulation and influences platelet activation. MAC can subsequently trigger the release of membrane microparticles from platelets and endothelial cells, increasing prothrombinase activity and coagulation through exposure of Fva. FIIa can cleave C3 and C5, thereby increasing complement activation through a positive feedback loop^17^. | Inhibition of complement system activation via the alternative pathway ^10^. | | | The venom of *B. lanceolatus* activates the complement system and decreases the lytic activity of the alternative pathway in a dose-dependent manner. It induces a significant production of C5a, capable of causing an influx of calcium into monocytes. However, the inhibition observed in the presence of 1,10-phenanthroline suggests that C5 cleavage and the associated release of C5a are mediated by the action of metalloproteases contained in the venom. C5b recruits C6, then C7, C8, and several C9 proteins to form the MAC (or complement terminal complex), a membrane pore that causes cell lysis. ^10^.  PLA2 from *B. lanceolatus* activates the complement system by releasing C5a and C3a^8^. |
| **Platelet-monocyte interaction** | P-selectin from alpha granules moves to the surface of the platelet and binds to PSGL1 located on the surface of monocytes. This interaction is then consolidated by several molecular bonds, notably between CD40L and monocyte CD40, between GPVI and extracellular matrix metalloproteinase inducer, and between GPIb-MAC-1. Platelets also attract monocytes by releasing chemokines and cytokines from their granules, such as CD40L, CXCL4, and CCL5^9^. | Null | | Studies have shown that *B. jararaca* significantly increases P-selectin on circulating platelets in venom-injected rabbits ^22^ and that jararhagin, a metalloproteinase in *B. jararaca* venom, can increase the number of rolling monocytes in mouse muscle venules ^23^. | Null |
| **Phagocytosis** | Phagocytosis occurs when the pathogen is recognized by opsonins, which bind via FcyR and C3bR receptors ^18^ leading to the internalization of the pathogen by endocytosis in neutrophils or macrophages (after differentiation of the monocyte), which are then destroyed in the phagosome^24^. | Null | | HF3, a PIII SVMP extracted from the venom of *B. jararaca*, triggered phagocytosis of opsonized zymosan particles by macrophages via integrin αmβ^4^. | Under the effect of the venom, the complement system induces opsonization.^10^. |
| **Degranulation and respiratory burst** | Degranulation involves the secretion of myeloperoxidase, neutrophil elastase, cytokines, and defensins by neutrophils (most commonly), monocytes, macrophages, NK cells, or other immune cells. Activation of the type 2 NADPH oxidase enzyme system leads to the rapid formation of reactive oxygen species (H₂O₂, O₂⁻) and then nitrogen (NO), all of which are powerful bactericides. The waste products are then released into the environment or used to activate adaptive immunity. This function is particularly effective in neutrophils^18^. | Faced with snake venom, neutrophils generate ROS, produce various pro-inflammatory cytokines and eicosanoids, and release NETs to defend the host ^25^. | | | |
| **NETosis** | Neutrophils release chromatin networks into the extracellular space, which are neutrophil extracellular traps (NETs). These NETs contain various pro-inflammatory and procoagulant components, including nuclear DNA, histones, myeloperoxidase, and neutrophil elastase. Negatively charged extracellular DNA activates FXII and thus initiates the intrinsic coagulation pathway, while histones promote platelet aggregation and activation. NETs also serve as a substrate for platelet binding. Neutrophil elastase released by NETs degrades TFPI, a coagulation inhibitor, while neutrophils-associated platelets reinforce this process and NET formation. Thus, neutrophil-platelet complexes promote tissue factor activity and stimulate coagulation.^26^.  NETosis is a form of programmed cell death in neutrophils, associated with the formation of NETs in response to various infectious or sterile stimuli (such as activated platelets, endothelial cells, cytokines, etc.). NETs have prothrombotic effects, promoting platelet activation and clot formation. When NETs are released, the neutrophil membrane dissolves, allowing decondensed chromatin and granular proteins to pass into the extracellular space. This process allows the neutrophil to trap and destroy pathogens through the release of its intracellular components ^27^. | BaTX-II, PLA2 from *B. atrox*, induces the release of double-stranded DNA from neutrophils of healthy donors^28^.  A study has shown that increased plasma levels of TF antigen correlate with hemostatic components (coagulation factors, platelets, and the fibrinolysis system), and that patients with systemic bleeding and those with moderate/severe edema have significantly higher TF levels^10^. | | It has been demonstrated that local or systemic administration of B. jararaca venom in mice increases plasma TF activity, accompanied by an increase in its expression in the skin and lungs. SVMPs, the main toxins in this venom, play a major role in this activation^10^. | NETosis is induced by the complement system ^10^. |

**References of Supplemental Table 2**

1. Espinosa É, Chillet P. *Immunologie*. ELLIPSES.; 2010.

2. De Toni LGB, Menaldo DL, Cintra ACO, et al. Inflammatory mediators involved in the paw edema and hyperalgesia induced by Batroxase, a metalloproteinase isolated from *Bothrops atrox* snake venom. *International Immunopharmacology*. 2015;28(1):199-207. doi:10.1016/j.intimp.2015.06.001

3. Sartim MA, Riul TB, Del Cistia-Andrade C, et al. Galatrox is a C-type lectin in Bothrops atrox snake venom that selectively binds LacNAc-terminated glycans and can induce acute inflammation. *Glycobiology*. 2014;24(11):1010-1021. doi:10.1093/glycob/cwu061

4. Teixeira C, Fernandes CM, Leiguez E, Chudzinski-Tavassi AM. Inflammation Induced by Platelet-Activating Viperid Snake Venoms: Perspectives on Thromboinflammation. *Front Immunol*. 2019;10. doi:10.3389/fimmu.2019.02082

5. Clissa PB, Laing GD, Theakston RDG, Mota I, Taylor MJ, Moura-da-Silva AM. L’effet de la jararhagine, une métalloprotéinase du venin de *Bothrops jararaca*, sur les cytokines pro-inflammatoires libérées par les cellules adhérentes péritonéales murines. *Toxicon*. 2001;39(10):1567-1573. doi:10.1016/S0041-0101(01)00131-3

6. Gallagher P, Bao Y, Serrano SMT, et al. Role of the snake venom toxin jararhagin in proinflammatory pathogenesis: In vitro and in vivo gene expression analysis of the effects of the toxin. *Archives of Biochemistry and Biophysics*. 2005;441(1):1-15. doi:10.1016/j.abb.2005.06.007

7. Arruda VA, Guimarães A de Q, Hyslop S, Araújo PMF de, Bon C, Araújo AL de. Le venin de *Bothrops lanceolatus* (Fer de lance) stimule la migration des leucocytes dans la cavité péritonéale des souris. *Toxicon*. 2003;41(1):99-107. doi:10.1016/S0041-0101(02)00238-6

8. Gabrili JJM, Pidde G, Magnoli FC, et al. New Insights into Immunopathology Associated to Bothrops lanceolatus Snake Envenomation: Focus on PLA2 Toxin. *Int J Mol Sci*. 2023;24(12):9931. doi:10.3390/ijms24129931

9. Rolling CC, Barrett TJ, Berger JS. Platelet-monocyte aggregates: molecular mediators of thromboinflammation. *Frontiers in Cardiovascular Medicine*. 2023;10:960398. doi:10.3389/fcvm.2023.960398

10. Cavalcante JS, Almeida DEG de, Santos-Filho NA, et al. Crosstalk of Inflammation and Coagulation in Bothrops Snakebite Envenoming: Endogenous Signaling Pathways and Pathophysiology. *International Journal of Molecular Sciences*. 2023;24(14):11508. doi:10.3390/ijms241411508

11. Santos BF, Serrano SMT, Kuliopulos A, Niewiarowski S. Interaction of viper venom serine peptidases with thrombin receptors on human platelets. *FEBS Letters*. 2000;477(3):199-202. doi:10.1016/S0014-5793(00)01803-2

12. Resiere D, Arias AS, Villalta M, et al. Évaluation préclinique de la capacité neutralisante d’un antivenin monospécifique pour le traitement des empoisonnements par *Bothrops lanceolatus* en Martinique. *Toxicon*. 2018;148:50-55. doi:10.1016/j.toxicon.2018.04.010

13. Luka N, South K, Jones R, et al. The Role of the VWF/ADAMTS13 Axis in the Thromboinflammatory Response in Ischemic Stroke After SARS‐CoV2 Infection. *Brain Behav*. 2025;15(2):e70348. doi:10.1002/brb3.70348

14. Serrano SMT, Wang D, Shannon JD, Pinto AFM, Polanowska-Grabowska RK, Fox JW. Interaction of the cysteine-rich domain of snake venom metalloproteinases with the A1 domain of von Willebrand factor promotes site-specific proteolysis of von Willebrand factor and inhibition of von Willebrand factor-mediated platelet aggregation. *FEBS J*. 2007;274(14):3611-3621. doi:10.1111/j.1742-4658.2007.05895.x

15. Thomazini CM, Soares R de PS, da Rocha TRF, Sachetto ATA, Santoro ML. Optimization of von Willebrand factor multimer analysis in vertical mini-gel electrophoresis systems: A rapid procedure. *Thromb Res*. 2019;175:76-83. doi:10.1016/j.thromres.2019.01.018

16. Thomazini CM, Sachetto ATA, de Albuquerque CZ, et al. Involvement of von Willebrand factor and botrocetin in the thrombocytopenia induced by Bothrops jararaca snake venom. *PLoS Negl Trop Dis*. 2021;15(9):e0009715. doi:10.1371/journal.pntd.0009715

17. Szepanowski RD, Haupeltshofer S, Vonhof SE, Frank B, Kleinschnitz C, Casas AI. Thromboinflammatory challenges in stroke pathophysiology. *Semin Immunopathol*. 2023;45(3):389-410. doi:10.1007/s00281-023-00994-4

18. Association des enseignants d’hématologie, d’immunologie et de biothérapies des UFR de pharmacie coordonné par Claire POUPLARD et Paul ROUZAIRE. Immunité innée et inflammation. In: *Hématologie-Immunologie et Biothérapie*. 2ème édition. Collection Internat pharmacie dirigée par Sébactien FAure et Jean-Paul Belon. Elsevier Masson; 2024.

19. Radouani F, Jalta P, Rapon C, et al. The Contrasting Effects of Bothrops lanceolatus and Bothrops atrox Venom on Procoagulant Activity and Thrombus Stability under Blood Flow Conditions. *Toxins*. 2024;16(9):400. doi:10.3390/toxins16090400

20. Wellmann IAM, Ibiapina HNS, Sachett JAG, et al. Correlating Fibrinogen Consumption and Profiles of Inflammatory Molecules in Human Envenomation’s by Bothrops atrox in the Brazilian Amazon. *Front Immunol*. 2020;11:1874. doi:10.3389/fimmu.2020.01874

21. Zuliani JP, Diniz-Sousa R, da Silva Setubal S, Boeno CN, Lopes JA, Zamuner SR. Inflammatory effects of phospholipase A2s present in snake venom of the genus *Bothrops*. In: Chakraborti S, ed. *Phospholipases in Physiology and Pathology*. Academic Press; 2023:173-196. doi:10.1016/B978-0-323-95698-7.00009-7

22. Santoro ML, Sano-Martins IS. Platelet dysfunction during Bothrops jararaca snake envenomation in rabbits. *Thromb Haemost*. 2004;92(8):369-383. doi:10.1160/TH04-02-0120

23. Olaoba OT, Karina dos Santos P, Selistre-de-Araujo HS, Ferreira de Souza DH. Snake Venom Metalloproteinases (SVMPs): A structure-function update. *Toxicon X*. 2020;7:100052. doi:10.1016/j.toxcx.2020.100052

24. Frédéric Gros, Sylvie Fournel, Samuel Liégeois, Daniel Richard, Pauline Soulas-Sprauel. *Atlas d’immunologie. De La Détection Du Danger à l’immunothérapie.* Dunod, 2018.; 2018.

25. Zuliani JP, Soares AM, Gutiérrez JM. Polymorphonuclear neutrophil leukocytes in snakebite envenoming. *Toxicon*. 2020;187:188-197. doi:10.1016/j.toxicon.2020.09.006

26. Vagionas D, Papadakis DD, Politou M, Koutsoukou A, Vasileiadis I. Thromboinflammation in Sepsis and Heparin: A Review of Literature and Pathophysiology. *In Vivo*. 2022;36(6):2542-2557. doi:10.21873/invivo.12991

27. Almskog LM, Ågren A. Thromboinflammation vs. immunothrombosis: strategies for overcoming anticoagulant resistance in COVID-19 and other hyperinflammatory diseases. Is ROTEM helpful or not? *Front Immunol*. 2025;16. doi:10.3389/fimmu.2025.1599639

28. Setúbal S da S, Pontes AS, Nery NM, et al. Human neutrophils functionality under effect of an Asp49 phospholipase A2 isolated from Bothrops atrox venom. *Toxicon X*. 2020;6:100032. doi:10.1016/j.toxcx.2020.100032
